# A distinct *APC* pathogenic germline variant identified in a southern Thai family with familial adenomatous polyposis

**DOI:** 10.1186/s12920-021-00933-y

**Published:** 2021-03-19

**Authors:** Worrawit Wanitsuwan, Sukanya Vijasika, Pichai Jirarattanasopa, Sukanya Horpaopan

**Affiliations:** 1 Department of Surgery, Faculty of Medicine, Prince of Songkla University, Songkhla, 90110 Thailand; 2 Department of Ophthalmology, Faculty of Medicine, Prince of Songkla University, Songkhla, 90110 Thailand; 3 Department of Anatomy, Faculty of Medical Science, Naresuan University, Phitsanulok, 65000 Thailand

**Keywords:** Familial adenomatous polyposis, *APC*, Splicing deletion, Exon skipping

## Abstract

**Background:**

Familial adenomatous polyposis (FAP) is caused by pathogenic germline variants in the *APC* gene. To date, multiple pathogenic variants in coding regions, splice sites, and deep intronic regions have been revealed. However, there are still pathogenic variants that remain unidentified.

**Methods:**

Twenty-nine primer pairs flanking exons 2–16 (i.e., coding exons 1–15) of *APC* and their exon–intron junctions were used for germline pathogenic variant screening in Southern Thai patients with familial adenomatous polyposis (FAP). Transcription analysis was performed to confirm the pathogenicity of a splice site deletion of intron 10. Family members were interviewed for clinical histories. Blood samples were collected from 18 family members for a segregation study. Subsequently, clinical data of affected members were collected from the hospital databases.

**Results:**

We found a distinct heterozygous 16-bp deletion at the splice donor site of intron 10 leading to a skipping of exon 10 which was confirmed by transcript analysis (*APC*: c 1312 + 4_1312 + 19del, r.934_1312del). Predictive testing for the pathogenic *APC* variant in 18 of the proband’s family members (ten healthy and eight affected) from three generations showed the same heterozygous germline pathogenic variant in eight affected adult members (15–62 years old) and two children (7 and 10 years old). Seven of the ten carriers of the disease-causing variant had undergone colonoscopy, and colonic polyps were found in all cases, which confirmed the segregation of the inherited pathogenic variant. The phenotypic spectrum was found to vary within the family; and some affected family members exhibited extracolonic manifestations.

**Conclusions:**

To our knowledge, the pathogenic *APC* variant, c.1312 + 4_1312 + 19del, r.934_1312del, has not previously been reported. This study is one of the few reports describing the phenotypic consequences of a pathogenic *APC* variant in a high number of affected family members.

**Supplementary Information:**

The online version contains supplementary material available at 10.1186/s12920-021-00933-y.

## Background

Familial adenomatous polyposis (FAP; MIM# 175,100) is an autosomal dominant inherited disorder occurring in approximately 1% of all colorectal cancers (CRC). FAP is characterized by large numbers of adenomatous polyps in the colon and rectum at an early age of onset. Adenomas usually occur in the second decade of life (average onset age is 16 years) and will become symptomatic in the third decade of life. The lifetime risk of CRC development is 100% if polyps are not removed [1]. The average age of CRC diagnosis in untreated susceptible individuals is 39 years [2]. Attenuated FAP (AFAP) is a mild form of FAP with less than one hundred colorectal adenomas and/or with late onset of adenoma development. Usually, both adenoma formation and CRC will occur 10–15 years later in AFAP compared with the classical FAP [3].

FAP is caused by high-penetrant heterozygous pathogenic variants in the tumor suppressor gene (TSG) *APC* on chromosome 5q22. All main transcripts together encompass 18 exons, however, the main reference APC transcript (LRG_130t1; NM_000038.4) consists of 16 exons with the first exon being non-coding. The last exon 16 (coding exon 15) is the largest, encompasses the majority of the total coding region. To date, more than a thousand different *APC* pathogenic variants have been identified in FAP patients and have been reported in the Human Gene Mutation Database (HGMD). Most of the variants result in truncated proteins while some of them lead to aberrant splicing. Although correlations between germline *APC* genotypes and FAP phenotypes are well known, they still need to be delineated further.

In this study, we report a distinct deletion of a splice donor site of intron 10 which leads to a complete skipping of exon 10 (coding exon 9) in a patient with FAP. Based on our segregation study and relevant clinical data, we found the pathogenic variant to be shared among all affected family members. We detail the phenotypic spectrum of affected members in this family. The supportive data confirm the pathogenicity of this variant. Our study is one of the few reports presenting the various phenotypes caused by the same variant in a large family.

## Methods

### *APC* genotyping study

Genomic DNA was extracted from peripheral blood leukocytes using the GeneJET Genomic DNA Purification kit (Thermo Scientific, Lithuania) according to manufacturer’s instructions. DNA samples were stored at − 20 °C until used.

Twenty-nine primer pairs flanking exons 2–16 (coding exons 1–15) of the *APC* gene and their exon–intron junctions were designed with Primer3Plus software (primer sequences are available upon request). PCR was performed following standard protocol using the TopTaq PCR master mix kit (Qiagen, Germany). The PCR product was purified with the QIAquick spin column (Qiagen, USA) and then cycle sequenced with the BigDye terminator kit version 2.0 and the ABI Prism 3500 Genetic Analyzer (Applied Biosystems).

### *APC* transcript analysis

We extracted total RNA from blood samples using the TRIzol reagent (Invitrogen, USA) and transcribed it into the first strand cDNA using the random hexamer and the SuperScript III First-Strand Synthesis System kit (Invitrogen, USA). The 647 cDNA base pairs exons 8 through 11 were amplified with specific primers. The sequences of forward and reverse primers were 5′-GGCAGAATGAAGGTCAAGGA-3′ and 5′- AACTAGGGGGACTACAGGCC-3′, respectively. We performed PCR following standard protocols and separated the PCR products on a 2% agarose gel. Deviant bands were excised, eluted, reamplified, and sequenced in both directions.

### Family screening

We interviewed the proband’s family members to obtain their family histories, and procured clinical data from the Hospital Information System (HIS) of the Songklanagarind University Hospital. Family members provided peripheral blood samples for germline *APC* pathogenic variant screening and transcription analysis. This study was approved by the Ethics Committees of Prince of Songkla University, and all patients gave informed consent.

## Results

### Case presentation

**Fig. 1:**
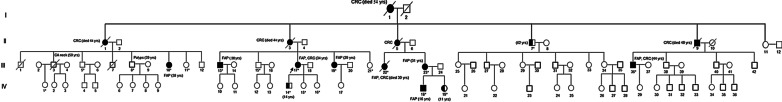
Pedigree 92 family members of a four-generations family from the southern Thailand; the black arrow denotes the proband. Nineteen family members including the proband, marked with an asterisk symbol, were screened the *APC* pathogenic variant c.1312 + 4_1312 + 19del, r.934_1312del. The proband and family members II:7, III:10, III:13, III:19, III:22, III:23, III:36, IV:14, IV:18, V:19 presented the germline pathogenic variant whereas the individuals III:5, III:8, III:11, III:15, III:21, IV:1, V:15, and IV:16 did not carry the pathogenic variant. Black shading family members were diagnosed CRC and/or FAP

The proband (case III:17) was a 29-year old female who presented symptoms with abdominal pain and mucus in bloody stool, weight loss of 10 kg within 4 months, and numerous adenomatous polyps (Fig. 1). Initial demographic data, clinical information, family history, results of endoscopic examinations, and laboratory data were obtained from the Hospital Information System (HIS). The patient provided full written informed consent.

**Fig. 2:**
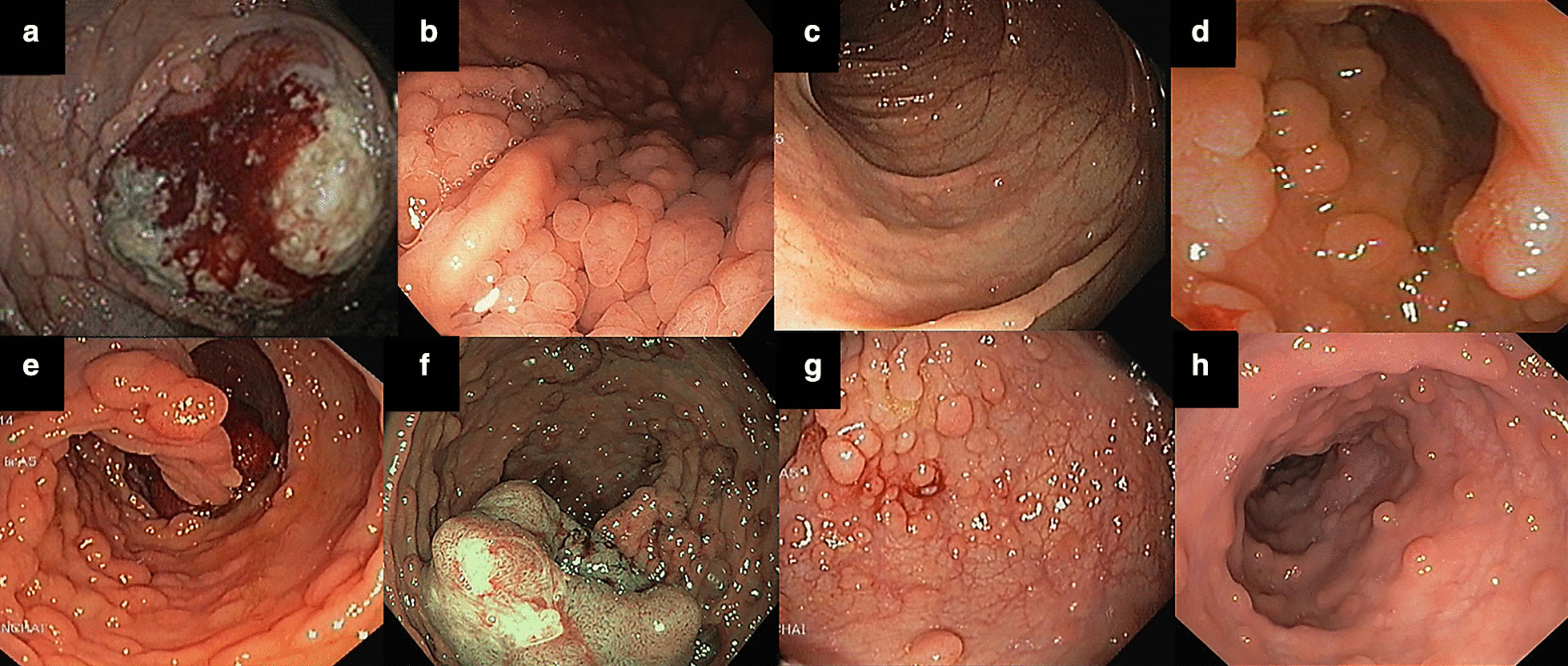
Colonoscopic screening of the proband (2a) and 7 family members (2b–2h) who carried the germline *APC* pathogenic variant (III:10, III:13, III:19, III:22, III:23, III:36 and IV:18)

A colonoscopy showed a sigmoid cancer with polyposis throughout the entire colon. Generally, the majority of polyps were 0.5–2.0 cm in diameter, sparing the area around the rectum (Fig. 2). A few polyps were up to 7 cm in diameter and were found in the transverse colon and the proximal rectum. In addition, a gastroscopy showed a few sessile polyps of 0.5–0.7 cm in the stomach.

In November of 2013, the patient obtained surgery for a total colectomy with ileorectal anastomosis. A histopathological report showed tumors of sizes up to 7 cm; a poorly differentiated adenocarcinoma; and metastatic adenocarcinoma in two of 37 nodes. After the operation, she received adjuvant chemotherapy with twelve cycles of FOLFOX-4 within six months, and then administration of Celebrex 400 mg daily for 1 year for the prevention of new polyps in the remaining part of the rectum. Subsequently, for follow-up purposes, she is having medical surveillance every six months. The latest chest and abdominal CT scan showed no recurrence of tumor, and no polyps were found at the anastomosis site and the rectum.

### *APC* pathogenic variant screening and transcription analysis

**Fig. 3:**
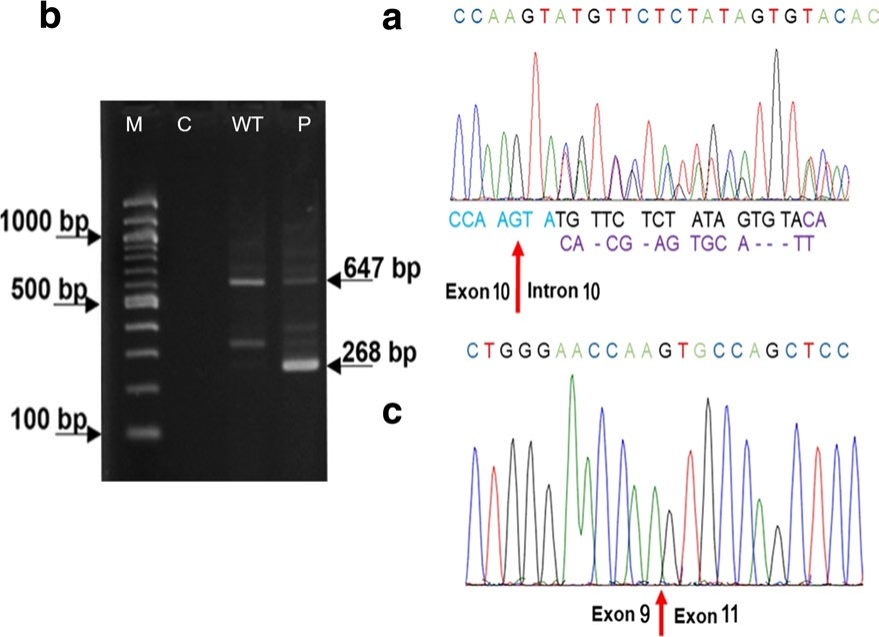
Genomic studies of carriers; **a** DNA sequencing of the exon–intron junction of exon 10 and intron 10 reveals a heterozygous germline deletion of the splice donor site of intron 10, c.1312 + 4_1312 + 19del, **b** Agarose gel showing RT-PCR products obtained from mRNA of the proband with primers localized in exon 8 (forward) and exon 11 (reverse), M is 100 bp ladder, C is negative control, WT is wild-type, and P is pathogenic variant. The expected size is 647 bp while the deletion size is 268 bp, **c** Sequencing pattern of the deviant band with 268 bp showing the junction of the 3′ end of exon 9 and the 5′ end of exon 11. The original uncropped gel is presented in supplementary Fig. S1

*APC* sequencing covering exon 10 and its splice regions showed a 16-bp deletion at the splice donor site of intron 10, c.1312 + 4_1312 + 19del in forward and reverse directions (Fig. 3a).

The transcript analysis covering exons 8–11 with 647 bp sequence length showed two dominant bands on the agarose gel, one of the size of the expected product with the other one being shorter (Fig. 3b). The sequencing of the shorter band showed a 379 bp deletion, a complete skipping of exon 10 of *APC*: r.934_1312del (Fig. 3c). The variant was submitted to the Leiden Open Variation Database (LOVD) at https://databases.lovd.nl/shared/individuals/ 00,324,918.

### Segregation analysis of *APC*: c.1312 + 4_1312 + 19del, r.934_1312del

Peripheral blood samples were obtained from 18 FAP family members (8 males and 10 females) for screening of the *APC* pathogenic variant at the splice donor site of intron 10 and a transcription analysis. To ascertain inheritance of the pathogenic variant, we were able to recruit family members from different generations; one from generation II, eleven from generation III, and six from generation IV.

The *APC* pathogenic variant, c.1312 + 4_1312 + 19del, r.934_1312del was detected in ten of the 18 members. Eight of ten had already been diagnosed with either FAP or CRC (Table 1, Fig. 1), Regardless, the other two members (IV:14 and IV:19) carried the pathogenic variant without displaying symptoms presumably because of their young ages of seven and ten years. The *APC* deletion was not found in the other eight members and these were healthy.

**Table 1:** Clinical data and histopathological data of the proband and family members

ID	Age (years)	Diagnosis	Age at diagnosis	APC pathogenic variant	Colonic manifestation		Extracolonic manifestation	
					Colonic polyps	Rectum polyps	CHRPE	Gastric polyps
II:7-^a^	> 60	FAP	62	Yes	N/A	N/A	N/A	N/A
III:5	51–60	Normal	48	No	N/A	N/A	N/A	N/A
III:8	41–50	Normal	40	No	N/A	N/A	N/A	N/A
III:10	41–50	FAP	38	Yes	> 1000	< 20	None	None
III:11	31–40	Normal	**/**	No	N/A	N/A	N/A	N/A
III:13	41–50	AFAP	36	Yes	< 10	< 20	N/A	None
III:15	31–40	Normal	**/**	No	N/A	N/A	N/A	N/A
III:17^**pro**^	31–40	Sigmoid CA	30	Yes	> 1000	< 20	None	Gastric polyps
III:19	21–30	FAP	24	Yes	> 1000	< 20	None	Gastric polyps
III:21	21–30	Normal	**/**	No	N/A	N/A	N/A	N/A
III:22^b^	Deceased	Rectal CA	29	Yes	> 1000	< 20	N/A	Gastric polyps
III:23	31–40	FAP	31	Yes	> 1000	< 20	None	Gastric polyps
III:36	41–50	Sigmoid CA	44	Yes	> 1000	< 20	None	Gastric polyps
IV:1	31–40	Normal	**/**	No	N/A	N/A	N/A	N/A
IV:14^**c**^	< 20	Normal	10	Yes	N/A	N/A	N/A	N/A
IV:15	< 20	Normal	**/**	No	N/A	N/A	N/A	N/A
IV:16	< 20	Normal	**/**	No	N/A	N/A	N/A	N/A
IV:18	< 20	FAP	15	Yes	> 100	< 20	None	Gastric polyps
IV:19^c^	< 20	Normal	7	Yes	N/A	N/A	None	N/A

^**a**^, II:7 can’t follow up; ^**b**^, III:22 was deceased prior to the present study; ^**c**^, IV:14 and IV:19 were too young for the colonoscopy; ^pro^, proband; AFAP, attenuated familial adenomatous polyposis; CHRPE, congenital hypertrophy of the retinal pigment epithelium; CRC, colorectal cancer; F, female; FAP, familial adenomatous polyposis; M, male; N/A, not applicable due to neither the colonoscopy nor EGD was not performed

### Family screening

We ascertained family histories of 92 family members in four generations by interviewing family members of the proband. The proband’s grandmother (I:1) and mother (II:3) were deceased with colon cancer at the age of 54 and 44, respectively. Similarly, her two aunts (II:1, II:5) and her uncle (II:9) had died with CRC (unknown age of death). The other uncle (II:7) was diagnosed with FAP at the age of 62. One of her cousins, III:3 developed neck cancer and died at the age of 50 (Fig. 1).

Based on the clinical histories and genetic testing, we collected detailed clinical data of seven family members carrying the pathogenic *APC* variant. The colonoscopic screening revealed varying numbers of colonic polyps (Fig. 2). However, three family members who carried the pathogenic variant did not have a colonoscopy: two of the three family members were too young at that time (IV:14 and IV:19) while the other was lost to follow-up. (II:7).

Of the ten carriers, III:10, III:19, and III:23, who were diagnosed at 38, 34, and 31 years of age, respectively, and each had more than 1000 colorectal polyps. All had undergone a total colectomy with ileorectal anastomosis (IRA). They have been followed up every six months and no polyps at the anastomosis site and in the rectum have been observed. Individual III:22 developed colon cancer in the rectum at the age of 29 years. The esophagogastroduodenoscopy (EGD) showed polyps (Spielman stage II) in the stomach and duodenum. Colonoscopy showed a 4.5 cm tumor at the lower rectum and more than 1000 polyps in the entire colon. She received a laparoscopic abdominoperineal resection (APR) with end ileostomy, which was followed by adjuvant chemotherapy treatment. After the absence of a follow-up for one year, she returned to the hospital with severe headaches that had been troubling her for two months. Her eyes presented with different conjunctival injections. The brain CT scan showed progression of a right petroclival metastasis. She died within 2–3 months. Individual III:36 was diagnosed with cancer in the sigmoid colon at 44 years of age. He presented with a change in bowel habit and rectal bleeding for a year. Colonoscopy showed a sigmoid colon mass and numerous (> 1000) polyps throughout the entire colon, however rectum sparingly. A CT scan of the chest and abdomen showed metastases in multiple organs including the lungs, liver, mesenteric lymph nodes, and the left adrenal gland. He had received palliative chemotherapy with FOLFOX-4 since June 2019. He died eight months later. Individual III:13 was diagnosed with AFAP at the age of 36. Colonoscopy exhibited a few tubular adenoma polyps in the rectum. At present, he has been taking Celebrex 400 mg daily for three years in combination with a surveillance colonoscopy every 1–2 years. One young patient, IV:18, aged 15 years, was diagnosed with FAP showing a few hundred polyps throughout the intestine. Pathological studies of a sigmoid colon biopsy and a gastric mucosa biopsy demonstrated a tubular adenoma and adenomatous polyps with low grade dysplasia. He is now obtaining a surveillance colonoscopy twice a year and will be subjected to a total colectomy at the age of 20. A complete fundus examination and re-checking with a wide field fundus photo showed no evidence of congenital hypertrophy of the retinal pigment epithelium (CHRPE) in all 7 family members. No other extra-intestinal manifestations including desmoid tumors, osteoma, papillary thyroid carcinoma, or hepatoblastoma were found in any of these family members. The clinical data and histopathologic diagnosis are listed in Table 1.

## Discussion

In this study, we identified a distinct germline pathogenic variant of the *APC* gene in a patient with FAP. The 16-bp deletion at the splice donor site of intron 10 (*APC*: c.1312 + 4_1312 + 19del), leads to a complete skipping of exon 10 (coding exon 9) of *APC*, which results in a shortening of the *APC* mRNA, r.934_1312del. We studied the segregation of the pathogenic variant among many affected and unaffected family members and examined genotype–phenotype correlations. We found that variant to be strongly associated with FAP. It was detected in all clinically affected family members, yet none of their relatives, in whom the variant was excluded, showed evidence for FAP-related symptoms. Only the youngest two pathogenic variant carriers have had no colonoscopy so far. The members of the family are from three generations, and the variant segregated with the disease phenotype. Both, the results of transcript analysis, demonstrating exon skipping, and the segregation with affected family members confirm that the variant is pathogenic and thus causes the phenotype for all variants carriers in the family.

Usually, the pathogenic relevance of a genetic variant has been evaluated according to its effect on the protein. Variants at the consensus splice acceptor or splice donor sites are predicted to abolish the splice recognition site and result in exon skipping. Disease-causing variants at the splice donor site of intron 10 leading to a skipping of exon 10 have been reported previously in the international *APC* reference database (www.lovd.nl/APC) [4, 5] but all those previously reported events are point mutations, small insertions, or large duplications. Therefore, to our knowledge, this heterozygous 16 bp deletion is a novel pathogenic splice variant resulting in the deletion of exon 10. We are able to demonstrate by cDNA analysis that this splice donor site variant leads to a complete deletion of the corresponding exon. Although we cannot completely rule out an mRNA nonsense mediated decay, previous reports indicated that aberrant APC transcripts are stable in general and do not significantly increase NMD [5–8].

In-silico analysis with the ExPASy Bioinformatics Resource Portal [9] predict an amino acid change due to deletion of the entire exon 10 (379 bp) resulting in a frameshift leading to a premature stop codon in exon 11 (*APC*: p.Val312CysfsTer15). A different splice donor variant resulting in the same frameshift variant has previously been reported [10]. This mutation leads to a lack of all functional domains, retaining only the homo-dimerization domain. Because the majority of the total coding region is located in exon 16, the largest exon, loss of APC function results in increased level of β-catenin and activation of growth-promoting genes via the downstream T-cell transcription factor (Tcf) pathways, that subsequently lead to the development of adenomatous colorectal polyps at a young age [11].

The typical colorectal phenotype with the formation of polyps starting during the second decade of life, has been reported in classical FAP associated with germline *APC* pathogenic variants in codons 157–1595, excluding variants in the cluster region (codon 1250–1464) which are found in FAP patients with a severe, early-onset colorectal phenotype [12]. *APC* pathogenic variants around exon 10 are known to be associated with classic FAP [13]. However, pathogenic variants in the alternative spliced region of exon 10 (codon 312–412) usually lead to an AFAP phenotype instead [14, 15]. In this study, one of the variant carriers (first-degree relative; III:13), exhibits an attenuated colorectal polyposis phenotype with less than one hundred polyps although we would expect a more typical FAP. Nevertheless, there have been a few studies supporting the notion that the skipping of exon 10 caused by a substitution at the splice donor site of intron 10 can lead to both attenuated and classic FAP [5, 6, 8].

Broad clinical variability is a well-known feature in FAP. In our study, the age of onset is varied (15–62 years.), the number of colonic polyps is also varied, from < 100 to > 1000. The majority of affected cases presented with more than a thousand colonic polyps and yet less than 20 polyps in the rectum. It is well known that the severity and the age of onset of FAP can vary considerably even within a family where all carriers harbor the same pathogenic variant. CHRPE and desmoid tumors are common extra-colonic manifestations [12, 16]. CHRPE is mainly associated with pathogenic variants between codons 463–1387 while desmoid tumors are in particular correlated with pathogenic variants between codons 1445–1578 [17, 18]. According to the location of the pathogenic variant identified in this family (codon 312), our result is consistent with the established genotype–phenotype correlation where neither CHRPE nor desmoid tumors are expected. However, there also might be other effects such as modifying genes or environmental factors contributing to the marked clinical variability of FAP even within families [5]. Additionally, a study in a large Chinese family with FAP reportedly identified the novel frameshift mutation c.1317delA, p.(Ala440LeufsTer14) in exon 11 of the *APC* gene leading to a change in protein [19]. These authors noticed that the termination in the exon 11 was correlated with extra colonic manifestations which includes duodenal polyposis and sebaceous cysts. In our study, we found multiple gastric polyps in six of eight carriers, including the proband who had undergone EGD, which is in line with previous studies showing that the gastroduodenal adenoma is related to the variants of *APC* in codons 564–1493 [12]. However, in our study, we did not find any hepatoblastoma, which might occur in FAP patients carrying the *APC* variant in codons 141–1751 [12]. These tumors should be added to the national surveillance program.

It is known that people with a heterozygous germline variant of *APC*, without early detection and prevention, would eventually develop FAP and CRC in late childhood or later. Fortunately, we detected the inherited pathogenic variant in two adolescents in this family. It is generally recommended that carriers should undergo regular surveillance and laparoscopic screening starting at age 12. In addition, all cases with the *APC* pathogenic variant, who already had developed FAP or CRC, were followed up for prophylactic management by a total colectomy with ileorectal anastomosis (IRA). Afterward, sigmoidoscopy has been scheduled every six months. As we lost contact with one family member (II:7) carrying the pathogenic variant of *APC*, we were unable to provide genetic screening and counselling for his children and grandchildren. Although these interventions significantly prevent CRC development, missing personal contact data and lack of patient co-operation are often barriers to systematic improvement of hereditary cancer surveillance.

## Conclusions

In summary, we identified in a multi-generation family with FAP a distinct germline splicing variant c.1312 + 4_ 1312 + 19del, r.934_1312del, p.(Val312CysfsTer15) in the *APC* gene (NM_000038.4). We confirmed its pathogenicity by co-segregation analysis and also found a wide range in the number of polyps in the family. Varying phenotypes are present ranging from AFAP through FAP with and without extracolonic manifestation. This study is one of the few reports able to document the phenotypic consequences of an *APC* pathogenic variant in a high number of affected family members. There seem to be various effects including environmental and genetic background factors resulting in considerable inter- and intrafamilial variability of the colorectal phenotype in *APC*-related FAP. Predictive genetic testing in early adolescence and intense surveillance are needed to provide pre-symptomatic diagnosis and a proper prevention before cancer development.

## Supplementary Information

**Additional file1: Supplementary Fig. S1**. The original agarose gel of the Fig. 3b, showing RT-PCR products obtained from mRNA of the proband.

## Data Availability

https://databases.lovd.nl/shared/individuals/00324918

## Abbreviations

AFAP: Attenuated familial adenomatous polyposis
APC: Adenomatous polyposis coli
APR: Abdominoperineal resection
bp: Base pair
cDNA: Complementary DNA
CHRPE: Congenital hypertrophy of the retinal pigment epithelium
cm: Centimeter
CRC: Colorectal cancer
CT: Computerized tomography
DNA: Deoxyribonucleic acid
EGD: Esophagogastroduodenoscopy
FAP: Familial adenomatous polyposis
HIS: Hospital information systems
IRA: Ileorectal anastomosis
mg: Milligram
mRNA: Messenger ribonucleic acid
PCR: Polymerase chain reaction
RNA: Ribonucleic acid
Tcf: T-cell transcription factor
TSG: Tumor suppressor gene
yrs: Years
