# Supplementary material for: A distinct APC pathogenic germline variant identified in a southern Thai family with familial adenomatous polyposis
Source: BMC Med Genomics. 2021 Mar 19;14:87. doi: 10.1186/s12920-021-00933-y (PMC7980625; doi:10.1186/s12920-021-00933-y)

Supplementary Fig. S1 The original agarose gel of the Fig. 3b, showing RT-PCR products obtained from mRNA of the proband.

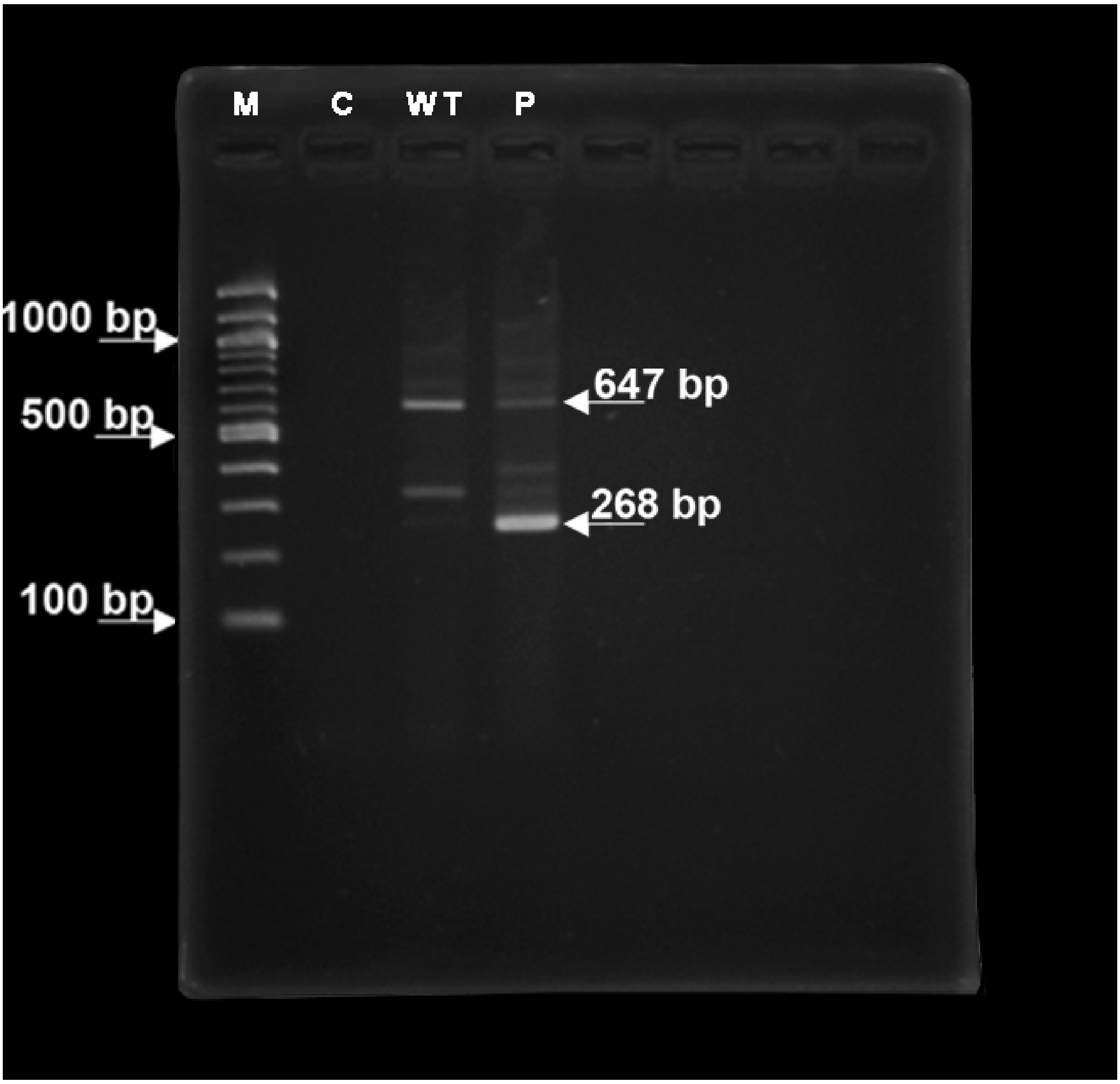

Supplement: Supplementary file 1 — Additional file1: Supplementary Fig. S1. The original agarose gel of the Fig. 3b, showing RT-PCR products obtained from mRNA of the proband. [file 12920_2021_933_MOESM1_ESM.pdf]
